# Case report: Compound heterozygous variants detected by next-generation sequencing in a Tunisian child with ataxia-telangiectasia

**DOI:** 10.3389/fneur.2024.1344018

**Published:** 2024-05-31

**Authors:** Nihel Ammous-Boukhris, Rania Abdelmaksoud-Dammak, Dorra Ben Ayed-Guerfali, Souhir Guidara, Olfa Jallouli, Hassen Kamoun, Chahnez Charfi Triki, Raja Mokdad-Gargouri

**Affiliations:** ^1^Laboratory of Eukaryotes’ Molecular Biotechnology, Center of Biotechnology of Sfax, University of Sfax, Sfax, Tunisia; ^2^Department of Human Genetics, Hedi Chaker Hospital, Sfax, Tunisia; ^3^Department of NeuroPediatry, Hedi Chaker Hospital, Sfax, Tunisia

**Keywords:** ataxia-telangiectasia, ATM, mutations, next generation sequencing, targeted sequencing

## Abstract

Ataxia-telangiectasia (A-T) is an autosomal recessive primary immunodeficiency disorder (PID) caused by biallelic mutations occurring in the serine/threonine protein kinase (*ATM*) gene. The major role of nuclear *ATM* is the coordination of cell signaling pathways in response to DNA double-strand breaks, oxidative stress, and cell cycle checkpoints. Defects in ATM functions lead to A-T syndrome with phenotypic heterogeneity. Our study reports the case of a Tunisian girl with A-T syndrome carrying a compound heterozygous mutation *c.[3894dupT]; p.(Ala1299Cysfs3;rs587781823)*, with a splice acceptor variant*: c.[5763-2A>C;rs876659489]* in the *ATM* gene that was identified by next-generation sequencing (NGS). Further genetic analysis of the family showed that the mother carried the c.[5763-2A>C] splice acceptor variant, while the father harbored the c.[3894dupT] variant in the heterozygous state. Molecular analysis provides the opportunity for accurate diagnosis and timely management in A-T patients with chronic progressive disease, especially infections and the risk of malignancies. This study characterizes for the first time the identification of compound heterozygous ATM pathogenic variants by NGS in a Tunisian A-T patient. Our study outlines the importance of molecular genetic testing for A-T patients, which is required for earlier detection and reducing the burden of disease in the future, using the patients’ families.

## Introduction

Ataxia-telangiectasia (A-T) is an autosomal recessive multisystem disorder characterized by progressive cerebellar degeneration, variable immunodeficiency, oculocutaneous telangiectasia, cancer susceptibility, and sensitivity to radiation (1, 2).

A-T patients represent a wide range of clinical manifestations, including progressive cerebellar ataxia, radiosensitivity, susceptibility to malignancies, and metabolic disorders. Other abnormalities, such as growth failure, poor pubertal development, insulin-resistant diabetes, gonadal atrophy, lung disease, cutaneous abnormality, and cardiovascular disease, have also been reported in A-T patients (3, 4). A-T patients have poor prognosis, and their survival time is approximately 25 years. The two most common causes of death in these patients are chronic pulmonary diseases and malignancy (5).

This syndrome is caused by biallelic pathogenic mutations in the ataxia-telangiectasia (*ATM*) gene containing 66 exons; of which, 62 are coding exons, spread over 150 kb of genomic DNA, with an open-reading frame of 9,168 nucleotides (6). This gene encodes a large protein (∼350 KDa) belonging to the phosphatidylinositol 3-kinase–related protein kinase (PIKK) family including *ATR*, *DNAPKcs*, *mTOR,* and *SMG1* genes (6, 7). As a member of the PIKK family, *ATM* contains a kinase domain positioned between conserved C-terminal domains known as FAT (FRAP, ATR, and TRRAP proteins), PIKK kinase, and FATC domains (7). These domains control ATM’s kinase activity by interacting with regulatory proteins and inducing posttranslational modifications (7).

ATM function is important to B-and T-cell receptor development and class switch recombination (CSR) in activated B cells (8). In addition, ATM plays a critical role in the repair of DNA double-strand breaks, the regulation of the cell cycle, the stability of the genome, and the survival of cells (9).

The majority of ATM pathogenic variants are single-nucleotide variant (SNV) alterations, such as frameshift or nonsense variants, which are predicted to truncate the ATM protein (8). Patients carrying these types of ATM mutations develop the classic form of A-T (10, 11).

Other SNV pathogenic variants of ATM include missense and splicing variants. According to the Human Gene Mutation Database, the copy number variation (CNV) or large genomics alterations are detected in approximately 1%–10% of A-T patients (12, 13). However, limited information is available on the co-occurrence of SNV and CNV and its identified role or phenotype burden in A-T patients (8).

This study reports for the first time a case of a Tunisian child diagnosed with A-T syndrome, who carried compound heterozygous ATM pathogenic variants, detected by targeted NGS. The co-segregation of both mutations was analyzed in the parents.

## Patient and methods

The proband in this study is a 16-year-old girl who had been followed up since the age of 6 years when she first presented with ocular telangectasia, foot drop, and proximo-distal deficit of both inferior extremities as addressed first to the Pediatric Neurology Department and then to Genetic Department of Hedi Chaker Hospital-Sfax Tunisia. The family pedigree information was gathered, and blood samples were collected from the patient and her parents. The proband had been the subject of various diagnostic tests, including magnetic resonance imaging (MRI) of the brain and cervical region, ultrasound examinations of the heart and abdomen, electroencephalogram (EEG), and blood biochemical analysis involving α-fetoprotein (AFP), immunoglobulin (Ig), and ceruloplasmin level detection.

Written informed consent was obtained from all participants.

### DNA extraction and targeted sequencing

The QIAamp DNA Blood Mini kit (Qiagen) was used to extract genomic DNA from 0.4 mL of peripheral blood obtained from the patient and her parents. The instructions of the manufacturer were followed during the extraction process. The resulting DNA was quantified using Qubit 3.0 (Thermo Fisher Scientific).

Briefly, 200 ng of genomic DNA was used to prepare the library using the OncoRisk panel kit, according to the protocol provided by Celemics. This panel includes 31 genes*: APC, ATM, BARD1, BLM, BMPR1A, BRCA1, BRCA2, BRIP1, CDH1, CDK4, CDKN2A, CHEK2, EPCAM, MLH1, MRE11A, MSH2, MSH6, MUTYH, NBN, PALB2, PMS2, PRSS1, PTEN, RAD50, RAD51C, RAD51D, SLX4, SMAD4, STK11, TP53, and VHL.*

Subsequently, the library was quantified with the Qubit® dsDNA HS Assay Kit (Life Technologies). The DNA library was pooled and prepared for sequencing using the MiSeq Reagent Kit v3 (300 cycles) according to the manufacturer’s instructions to generate paired-end reads with a read length of 151 bp (Illumina, San Diego, CA). Reads were trimmed to remove low-quality sequences and then aligned to the human reference genome (GRCh37/hg19) using the Burrows–Wheeler alignment (BWA) package. The ATM (NM_000051.3) sequence from the National Center for Biotechnology Information (NCBI) database^2^ was used as the reference, and NGS data were analyzed using the BaseSpace Variant Interpreter.^3^ SplicAI and SPIP prediction tools were used to evaluate the effect of the splice site acceptor variant (14, 15).

### Sanger sequencing

Sanger sequencing was used to confirm the presence of the variants identified by NGS and to investigate co-segregation analysis in the family members. Forward and reverse primers were designed using Primer 3.0 software to amplify the fragments covering the variant region and provided upon request. PCR products were purified and labeled using the BigDye Terminator V3.1 Cycle Sequencing Kit and sequenced on SeqStudio (Applied Biosystems). Sequence analysis was performed using BioEdit software.

## Results

### Case presentation

**Figure 1:**
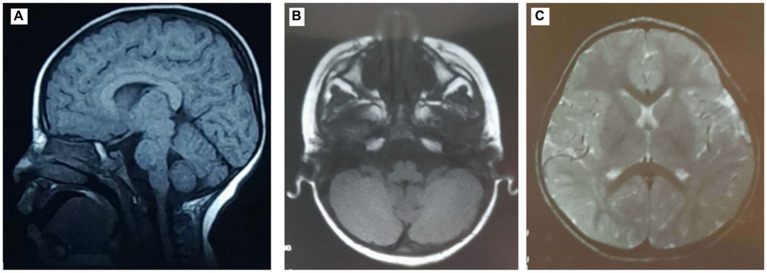
Magnetic resonance images (MRIs) of the patient’s brain showing discrete cerebellum atrophy. **(A)** Sagittal T1-weighted brain MRI. Axial T1- **(B)** and T2- **(C)** weighted brain MRI.

The proband *(IV-3)* is a 16-year-old girl who had no pre-, peri-, or post-natal complications and normal cognitive and motor development. She was consulted at the age of 6 years for abnormal movements and has since then followed up for cerebellar ataxia at the Department of Pediatric Neurology and Department of Genetics, at the CHU Hedi Chaker of Sfax, Tunisia. The proband *(IV-3)* had choreic abnormal movements affecting the upper and lower extremities since the age of 4.5 years, and upon examination, she had no facial dysmorphia, normal measurements, dysarthric speech, oculomotor apraxia, and static and kinetic cerebellar syndrome. She had difficulties at school, and due to worsening instability, she became bedridden at the age of 10 years. Ocular telangectasia was observed at 10 years of age, and after a year, she had developed foot drop and proximo-distal deficit of both inferior extremities and choreo-athetosis movements. Brain MRI performed at 3 years of age showed discrete cerebellum atrophy (Figure 1). EMG at the age of 12 years showed no neuropathy but was in favor of myoclonic dystonia. The EEG at the age of 11 years was well-organized, without abnormalities.

Concerning biochemical parameters, the serum alpha-FP levels were significantly increased from 125.2 ng/mL at the age of 6 years to 370 ng/mL at the age of 15 years, whereas the serum level of IgA was significantly decreased. Other biological analyses showed all normal levels of cholesterol, creatinine alkaline, lactate dehydrogenase (LDH), and ceruloplasmin.

The older sister (IV-2) experienced similar symptoms but showed a delay in language and walking ability. She also had tachycardia and suffered from immune deficiency, which was treated with venoglobulin transfusions. She died at the age of 16 years after a cardiac arrest.

**Figure 2:**
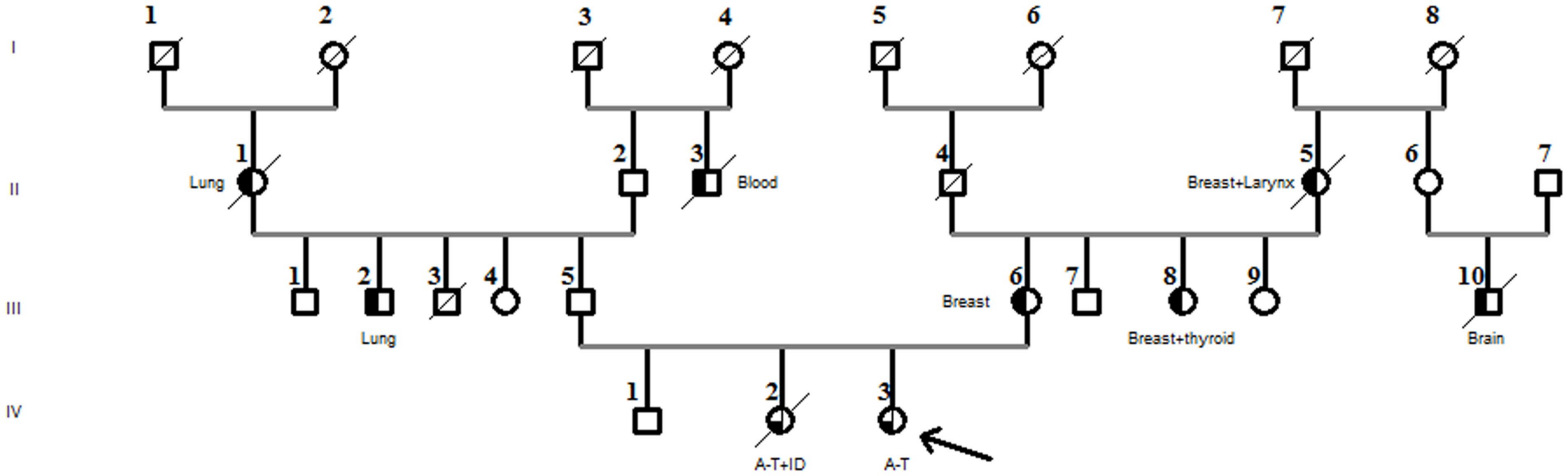
Family pedigree diagnosed with the compound heterozygous *ATM* pathogenic variant. The arrow in the pedigree member shows the A-T patient. The sister of the proband had A-T associated with immunodeficiency (A-T + ID). Black half-filled pedigree members indicate cancer cases (cancer types are mentioned in the pedigree).

There was no known consanguinity in the family, but there were a few cases of blindness. The proband’s mother *(III-6)* and aunt *(III-8)* both had breast cancer, and her paternal cousin was treated for autism (Figure 2).

### Genetic testing

The blood DNA of the proband was analyzed by NGS using a panel covering 31 genes (*Oncorisk* and *Celemics*) related to human malignancies. Approximately 99.9% of target regions were covered with at least 50X, and the mean region coverage depth was 3570.5. After filtering, two variants in the *ATM* gene, namely, NM_000051.3 *c.[3894dupT];p.(Ala1299Cysfs3;rs587781823)* and c.[5763-2A>C; *rs876659489*], were identified in the patient. According to the ClinVar database and ACMG criteria, the frameshift c.[3894dupT] is located in exon 26/63 and is classified as pathogenic (*class 5, PVS1, PM2,* and *PP5_Very Strong*). This mutation led to a frameshift at residue 1,299, which produced a truncated protein of 1,312 amino acids p. (Ala1299Cysfs3) lacking the FAT, PI3K/PI4K catalytic, and FATC domains. This variant is rare, with a population frequency equal to 0.00000796 (exomes) and 0.000163 (GnomAD).

On the other hand, the c.[5763-2A>C; *rs876659489*] variant is expected to disrupt RNA splicing by affecting an acceptor splice site in intron 38 of the *ATM* gene; thereby, it is classified as a class 5 pathogenic variant according to the ClinVar database and *ACMG* criteria (*PVS1_Moderate, PM2, PP3_Strong,* and *PP5_Very_Strong*). The *SpliceAI* and *SPIP* tools predicted that the *c.[5763-2A>C]* variant results in an acceptor loss with scores = 1 and −0.99, respectively. The population frequency of this variant is 0.000009, as indicated by GnomAD.

In addition, the proband *(IV-3)* carried six other variants in the *ATM* gene: one synonymous missense variant c.[5948A>G];p.(Ser1983=*rs659243*) and five intronic variants, all classified as benign. No other pathogenic variant has been identified in genes included in the NGS panel in the present study.

**Figure 3:**
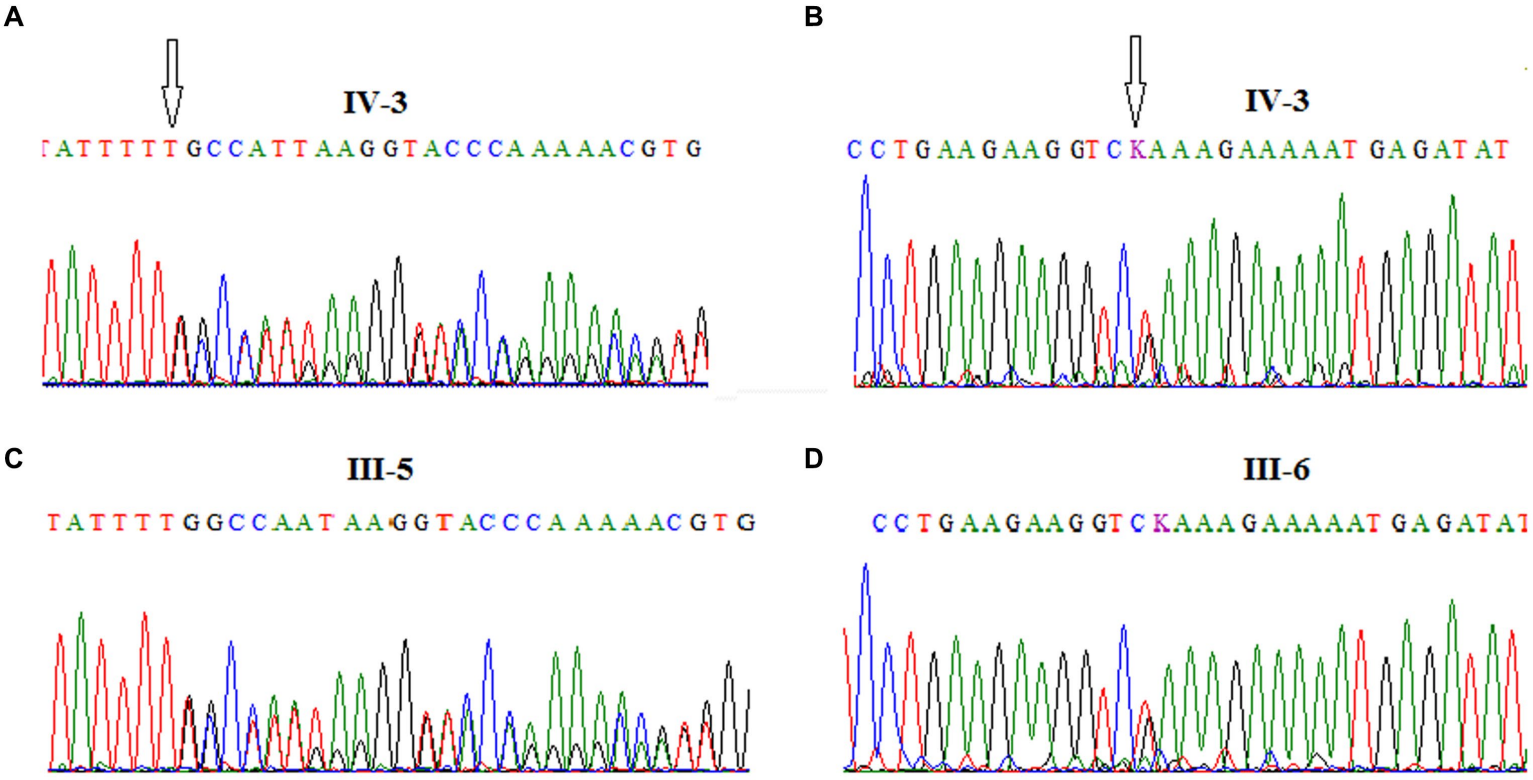
Chromatograms showing the frameshift mutation *c.[3894dupT]; p.*(*Ala1299CysfsTer3; rs587781823*), in ATM exon 26 identified in the A-T patient **(A)** and her father **(C)** and the splice site acceptor mutation *c.[5763-2A>C; rs876659489]* in ATM exon 39 identified in the A-T patient **(B)** and her mother **(D)**.

Furthermore, the DNA of the proband *(IV-3)* and her parents *(III-5 and III-6)* were subjected to Sanger sequencing to (i) confirm the variants found by NGS in the proband and (ii) investigate the heredity of both variants in the parents. Both variants were successfully verified in the proband; in addition, we found that the c.[3894dupT];p.(Ala1299CysfsTer3;*rs587781823*) variant, in the exon 26 of the *ATM* gene, was inherited from her father, and the c.[5763-2A>C *rs876659489*] splice site acceptor variant (intron 38) was inherited from her mother (Figure 3). It is important to mention that in these families, the first-degree relatives over two generations were affected by breast cancer, namely, the proband’s mother *(III-6)*, her aunt *(III-8)*, and her grandmother *(II-5)*, but unfortunately, their DNA samples were not available for genetic testing (Figure 2).

## Discussion

Ataxia-telangiectasia (A-T) is a rare disorder affecting multiple body systems. Typically, the degeneration of the nervous system begins between 6 and 18 months of age, resulting in being confined to a wheelchair by the age of 10 years. Cerebellar degeneration causes symptoms such as unsteady trunk movements, difficulty walking, lack of coordination, weak muscles, and sudden falls (16). The involuntary movements in A-T patients worsen over time, starting mildly in childhood and becoming more noticeable in adulthood. Movement disorders characterized by reduced movement are less common compared to those with excessive movement. Some patients might develop symptoms resembling Parkinson’s disease, such as stiffness and tremors when at rest (17).

It is well known that the mode of inheritance for A-T is autosomal recessive and caused by biallelic mutations in the *ATM* gene. The ATM protein plays a pivotal role in regulating several tumor suppressor proteins, mainly TP53, BRCA1, Chek2, RAD17, RAD9, and NBS1 (18, 19). These proteins, along with ATR kinase, are considered master controllers of cell cycle checkpoint signaling pathways, essential for the cell’s response to DNA damage and maintenance of genome stability (19, 20). Thus, when both copies of the *ATM* gene are inactivated (biallelic inactivation), it leads to A-T.

It is important to mention that in Tunisia, only two studies have investigated the clinical, immunological, and molecular (chromosomal instability) features without identifying the causal *ATM* gene mutation (21, 22). Therefore, our study is the first one that reports a Tunisian A-T patient harboring compound heterozygous mutations in the *ATM* gene, namely, a frameshift *c.[3894dupT];p.(Ala1299CysfsTer3;rs587781823)* and a splice site acceptor variant (*c.*[*5763-2A>C*]*,rs876659489*). According to the literature, the frameshift variant had been previously identified in a homozygous state in Italian and Polish A-T patients (23, 24).

Globally, most *ATM* gene mutations involve frameshift or nonsense mutations located in the proximal, central, and distal regions of the *ATM* gene (25). Barone et al. demonstrated that the majority of *ATM* missense mutations in A-T are functionally linked to defects in expression and/or inactivation of kinase activity (26). Additionally, Jacquemin et al. showcased that, aside from resulting in the under-expression of the ATM protein, ATM missense mutations caused abnormal cytoplasmic localization of the protein (27).

A recent study on Iranian A-T patients reported that ATM nonsense and frameshift mutations are most frequent, leading to a more severe phenotype than missense or splice-site mutations (28). However, in Chinese A-T patients, the mutational spectrum of ATM is likely to be diverse and different, when largely compared to other ethnic areas (29). Biallelic ATM mutations combining the splice site variant with frameshift, nonsense, or missense mutations were less frequent than other compound mutations. Despite this, there is a recent case report that described a Chinese A-T patient diagnosed at 7 years of age with the compound heterozygous ATM genotype (frameshift combined with splice site ATM variant), who is similar to the proband in our case (30). This Chinese girl presented with growth retardation, ataxia, medium ocular telangiectasia, cerebellar atrophy, elevated serum alpha-fetoprotein (AFP) level, and normal serum levels of immunoglobulins, which are all similar to our proband.

Altogether, our patient had an onset of A-T syndrome at the age of 6 years with slow progression and a lack of basal ganglia manifestations, ruling out immunodeficiency, which may indicate that her mutations led to less severe neurodegenerative effects compared to other mutations in the *ATM* gene.

Furthermore, there is increasing evidence showing that heterozygous mutations in the *ATM* gene are associated with an increased risk of developing a wide spectrum of malignancies, including breast, stomach, and lung cancers (31). We observed that the Tunisian family consists of several members with various types of cancer such as lung, larynx, brain, and breast. In line with this report, we confirmed that the proband’s mother, who had breast cancer, carried the pathogenic ATM variant *c.[3894dupT];p.*(*Ala1299CysfsTer3;rs587781823*), which is most likely responsible for the malignancy.

Although there is currently no cure for A-T patients, there has been a rapid development of mutation-targeted therapeutic approaches. These advancements bring hope for potential treatment in specific A-T patients with ATM mutations (32). These mutations can be corrected, for example, using antisense morpholino oligonucleotide (AMO), read through compound (RTC), or micro-RNA (33, 34). In fact, AMOs have shown effectiveness in correcting type II and IV splicing mutations (35). Research has also revealed that functional ATM protein can be induced using RTCs to target premature termination codons in cells with ATM heterozygous nonsense mutations (33). Furthermore, gene editing approaches, such as CRISPR/Cas9, have been employed for targeting the ATM gene, offering a promising tool for new therapeutic approaches in treating this disease (36).

These *in vitro* tests shed light on the potential therapeutic applications of customized mutation-targeted therapies for A-T patients in the future. However, it is very important to note that this personalized approach profoundly relies on an exhaustive analysis of ATM gene mutations.

## Conclusion

In summary, we report a case of an A-T patient carrying a compound heterozygous mutation c.[3894dupT];p.(Ala1299CysfsTer3*;rs587781823*) and c.[5763-2A>C *rs876659489*] splice acceptor variant in the *ATM* gene. Our findings extend the genotype spectrum of A-T in the Tunisian population and will allow timely decisions to be made in A-T diagnosis for better therapeutic management.

## Data availability statement

The datasets presented in this article are not readily available because of ethical and privacy restrictions. Requests to access the datasets should be directed to the corresponding author.

## Ethics statement

The studies involving humans were approved by the Comité de Protection des personnes CHU Hedi Chaker/CHU Habib Bourguiba sfax—Tunisia. The studies were conducted in accordance with the local legislation and institutional requirements. Written informed consent for participation in this study was provided by the participants' legal guardians/next of kin. Written informed consent was obtained from the individual(s), and minor(s)' legal guardian/next of kin, for the publication of any potentially identifiable images or data included in this article.

## Author contributions

NA-B: Investigation, Methodology, Writing – original draft, Writing – review & editing. RA-D: Data curation, Investigation, Methodology, Writing – review & editing. DB: Formal analysis, Methodology, Writing – review & editing. SG: Conceptualization, Formal analysis, Writing – review & editing. OJ: Formal analysis, Writing – review & editing. HK: Writing – review & editing. CC: Supervision, Validation, Writing – review & editing. RM-G: Conceptualization, Formal analysis, Supervision, Writing – original draft, Writing – review & editing.

## Footnotes

^2^ http://www.ncbi.nlm.nih.gov

^3^ https://basespace.illumina.com

## References

[1] Chaudhary MW Al-Baradie RS. Ataxia-telangiectasia: future prospects. Appl Clin Genet. (2014) 7:159–67. doi: 10.2147/TACG.S35759, PMID: 25258552 PMC4173637

[2] Nissenkorn A Levy-Shraga Y Banet-Levi Y Lahad A Sarouk I Modan-Moses D. Endocrine abnormalities in ataxia telangiectasia: findings from a national cohort. Pediatr Res. (2016) 79:889–94. doi: 10.1038/pr.2016.19 26891003

[3] Su Y Swift M. Mortality rates among carriers of ataxia-telangiectasia mutant alleles. Ann Intern Med. (2000) 133:770–8. doi: 10.7326/0003-4819-133-10-200011210-00009, PMID: 11085839

[4] Bott L Lebreton J Thumerelle C Cuvellier J Deschildre A Sardet A. Lung disease in ataxia-telangiectasia. Acta Paediatr. (2007) 96:1021–4. doi: 10.1111/j.1651-2227.2007.00338.x 17524020

[5] Crawford TO Skolasky RL Fernandez R Rosquist KJ Lederman HM. Survival probability in ataxia telangiectasia. Arch Dis Child. (2006) 91:610–1. doi: 10.1136/adc.2006.094268, PMID: 16790721 PMC2082822

[6] Gately DP Hittle JC Chan GK Yen TJ. Characterization of ATM expression, localization, and associated DNA-dependent protein kinase activity. Mol Biol Cell. (1998) 9:2361–74. doi: 10.1091/mbc.9.9.2361, PMID: 9725899 PMC25502

[7] Nanda N Roberts NJ. ATM serine/threonine kinase and its role in pancreatic risk. Genes. (2020) 11:108. doi: 10.3390/genes11010108, PMID: 31963441 PMC7017295

[8] Bakkenist CJ Kastan MB. DNA damage activates ATM through intermolecular autophosphorylation and dimer dissociation. Nature. (2003) 421:499–506. doi: 10.1038/nature01368, PMID: 12556884

[9] Aki T Uemura K. Cell death and survival pathways involving ATM protein kinase. Genes. (2021) 12:581. doi: 10.3390/genes12101581, PMID: 34680975 PMC8535589

[10] Verhagen MM Last JI Hogervorst FB Smeets DF Roeleveld N Verheijen F. Presence of ATM protein and residual kinase activity correlates with the phenotype in ataxia-telangiectasia: a genotype-phenotype study. Hum Mutat. (2012) 33:561–71. doi: 10.1002/humu.22016, PMID: 22213089

[11] Taylor AM Lam Z Last JI Byrd PJ. Ataxia telangiectasia: more variation at clinical and cellular levels. Clin Genet. (2015) 87:199–208. doi: 10.1111/cge.12453, PMID: 25040471

[12] Cavalieri S Funaro A Porcedda P Turinetto V Migone N Gatti RA. ATM mutations in Italian families with ataxia telangiectasia include two distinct large genomic deletions. Hum Mutat. (2006) 27:1061. doi: 10.1002/humu.9454, PMID: 16941484

[13] Cavalieri S Funaro A Pappi P Migone N Gatti RA Brusco A. Large genomic mutations within the ATM gene detected by MLPA, including a duplication of 41 kb from exon 4 to 20. Ann Hum Genet. (2008) 72:10–8. doi: 10.1111/j.1469-1809.2007.00399.x, PMID: 17910737

[14] Jaganathan K Kyriazopoulou Panagiotopoulou S McRae JF Darbandi SF Knowles D Li YI. Predicting splicing from primary sequence with deep learning. Cell. (2019) 176:535–48.e24. doi: 10.1016/j.cell.2018.12.015 30661751

[15] Leman R Parfait B Vidaud D Girodon E Pacot L Le Gac G. SPiP: splicing prediction pipeline, a machine learning tool for massive detection of exonic and intronic variant effects on mRNA splicing. Hum Mutat. (2022) 43:2308–23. doi: 10.1002/humu.24491, PMID: 36273432 PMC10946553

[16] Hoche F Seidel K Theis M Vlaho S Schubert R Zielen S. Neurodegeneration in ataxia telangiectasia: what is new? What is evident? Neuropediatrics. (2012) 43:119–29. doi: 10.1055/s-0032-1313915, PMID: 22614068

[17] Pearson TS. More than Ataxia: hyperkinetic movement disorders in childhood autosomal recessive Ataxia syndromes. Tremor Other Hyperkin Move. (2016) 6:368. doi: 10.5334/tohm.319 PMC4950223 27536460

[18] Zolotovskaia MA Modestov AA Suntsova MV Rachkova AA Koroleva EV Poddubskaya EV. Pan-cancer antagonistic inhibition pattern of ATM-driven G2/M checkpoint pathway vs other DNA repair pathways. DNA Repair. (2023) 123:103448. doi: 10.1016/j.dnarep.2023.103448, PMID: 36657260

[19] Phan LM Rezaeian AH. ATM: Main features, signaling pathways, and its diverse roles in DNA damage response, tumor suppression, and Cancer development. Genes. (2021) 12:845. doi: 10.3390/genes12060845, PMID: 34070860 PMC8228802

[20] Gatti RA Berkel I Boder E Braedt G Charmley P Concannon P. Localization of an ataxia-telangiectasia gene to chromosome 11q22-23. Nature. (1988) 336:577–80. doi: 10.1038/336577a0 3200306

[21] Triki C Feki I Meziou M Turki H Zahaf A Mhiri C. Clinical, biological and genetic study of 24 patients with ataxia telangiectasia from southern Tunisia. Rev Neurol. (2000) 156:634–7. 10891797

[22] Sfaihi L Stoppa Lyonnet D Ben Ameur S Dubois D'enghien C Kamoun T Barbouch MR. Ataxia-telangiectasia in the south of Tunisia: a study of 11 cases. La Tunisie Medicale. (2015) 93:511–5. PMID: 26815515

[23] Chessa L Piane M Magliozzi M Torrente I Savio C Lulli P. Founder effects for ATM gene mutations in Italian Ataxia telangiectasia families. Ann Hum Genet. (2009) 73:532–9. doi: 10.1111/j.1469-1809.2009.00535.x, PMID: 19691550

[24] Podralska MJ Stembalska A Ślęzak R Lewandowicz-Uszyńska A Pietrucha B Kołtan S. Ten new ATM alterations in polish patients with ataxia-telangiectasia. Mol Genet Genomic Med. (2014) 2:504–11. doi: 10.1002/mgg3.98, PMID: 25614872 PMC4303220

[25] Telatar M Teraoka S Wang Z Chun HH Liang T Castellvi-Bel S. Ataxia-telangiectasia: identification and detection of founder-effect mutations in the ATM gene in ethnic populations. Am J Hum Genet. (1998) 62:86–97. doi: 10.1086/301673, PMID: 9443866 PMC1376800

[26] Barone G Groom A Reiman A Srinivasan V Byrd PJ Taylor AM. Modeling ATM mutant proteins from missense changes confirms retained kinase activity. Hum Mutat. (2009) 30:1222–30. doi: 10.1002/humu.21034, PMID: 19431188

[27] Jacquemin V Rieunier G Jacob S Bellanger D d'Enghien CD Laugé A. Underexpression and abnormal localization of ATM products in ataxia telangiectasia patients bearing ATM missense mutations. Eur J Hum Genet. (2012) 20:305–12. doi: 10.1038/ejhg.2011.196, PMID: 22071889 PMC3283185

[28] Amirifar P Ranjouri MR Pashangzadeh S Lavin M Yazdani R Moeini Shad T. The spectrum of ATM gene mutations in Iranian patients with ataxia-telangiectasia. Pediatr Allergy Immunol. (2021) 32:1316–26. doi: 10.1111/pai.13461, PMID: 33547824

[29] Huang Y Yang L Wang J Yang F Xiao Y Xia R. Twelve novel Atm mutations identified in Chinese ataxia telangiectasia patients. NeuroMolecular Med. (2013) 15:536–40. doi: 10.1007/s12017-013-8240-3 23807571 PMC3732755

[30] Shao L Wang H Xu J Qi M Yu Z Zhang J. Ataxia-telangiectasia in China: a case report of a novel ATM variant and literature review. Front Neurol. (2023) 14:1228810. doi: 10.3389/fneur.2023.1228810, PMID: 37564729 PMC10411728

[31] Sriramulu S Ramachandran M Subramanian S Kannan R Gopinath M Sollano J. A review on role of ATM gene in hereditary transfer of colorectal cancer. Acta Biomed. (2019) 89:463–9. doi: 10.23750/abm.v89i4.6095, PMID: 30657113 PMC6502098

[32] Chessa LMR Molinaro A. Focusing new Ataxia telangiectasia therapeutic approaches. J Rare Dis Diagn. (2016) 2:2. doi: 10.21767/2380-7245.100041

[33] Nakamura K Du L Tunuguntla R Fike F Cavalieri S Morio T. Functional characterization and targeted correction of ATM mutations identified in Japanese patients with ataxia-telangiectasia. Hum Mutat. (2012) 33:198–208. doi: 10.1002/humu.21632, PMID: 22006793 PMC3261637

[34] Hu H Gatti RA. Micro RNAs: new players in the DNA damage response. J Mol Cell Biol. (2011) 3:151–8. doi: 10.1093/jmcb/mjq042, PMID: 21183529 PMC3104011

[35] Du L Gatti RA. Potential therapeutic applications of antisense morpholino oligonucleotides in modulation of splicing in primary immunodeficiency diseases. J Immunol Methods. (2011) 365:1–7. doi: 10.1016/j.jim.2010.12.001, PMID: 21147113 PMC3061259

[36] Nurieva W Ivanova E Chehab S Singh P Reichlmeir M Szuhai K. Generation of four gene-edited human induced pluripotent stem cell lines with mutations in the ATM gene to model Ataxia-telangiectasia. Stem Cell Res. (2023) 73:103247. doi: 10.1016/j.scr.2023.103247 37976651

